# Variants in the Toll-interacting protein gene are associated with susceptibility to sepsis in the Chinese Han population

**DOI:** 10.1186/cc9413

**Published:** 2011-01-10

**Authors:** Zhenju Song, Jun Yin, Chenling Yao, Zhan Sun, Mian Shao, Yaping Zhang, Zhengang Tao, Peizhi Huang, Chaoyang Tong

**Affiliations:** 1 Department of Emergency Medicine, Zhongshan Hospital, Fudan University, 180 Fenglin Road, Shanghai 200032, PR China

## Abstract

**Introduction:**

Deregulated or excessive host immune responses contribute to the pathogenesis of sepsis. Toll-like receptor (TLR) signaling pathways and their negative regulators play a pivotal role in the modulation of host immune responses and the development of sepsis. The objective of this study was to investigate the association of variants in the TLR signaling pathway genes and their negative regulator genes with susceptibility to sepsis in the Chinese Han population.

**Methods:**

Patients with severe sepsis (*n *= 378) and healthy control subjects (*n *= 390) were enrolled. Five genes, namely *TLR2, TLR4, TLR9, MyD88 *and *TOLLIP*, were investigated for their association with sepsis susceptibility by a tag single nucleotide polymorphism (SNP) strategy. Twelve tag SNPs were selected based on the data of Chinese Han in Beijing from the HapMap project and genotyped by direct sequencing. The mRNA expression levels of *TOLLIP *were determined using real-time quantitative Polymerase Chain Reaction (PCR) assays, and concentrations of tumor necrosis factor alpha (TNF-α) and interleukin-6 (IL-6) were measured by enzyme-linked immunosorbent assay (ELISA).

**Results:**

Our results showed that the minor C-allele of rs5743867 in *TOLLIP *was significantly associated with the decreased risk of sepsis (*P*_*adj *_= 0.00062, odds ratio (OR)_*adj *_= 0.71, 95% confidence interval (CI) 0.59 to 0.86) after adjustment for covariates in multiple logistic regression analysis. A 3-SNP haplotype block harboring the associated SNP rs5743867 also displayed strong association with omnibus test *P *value of 0.00049. Haplotype GTC showed a protective role against sepsis (*P*_*adj *_= 0.0012), while haplotype GCT showed an increased risk for sepsis (*P*_*adj *_= 0.00092). After exposure to lipopolysaccharide (LPS), *TOLLIP *mRNA expression levels in peripheral blood mononuclear cells (PBMCs) from homozygotes for the rs5743867C allele were significantly higher than in heterozygotes and homozygotes for the rs5743867T allele (*P *= 0.013 and *P *= 0.01, respectively). Moreover, the concentrations of TNF-α and IL-6 in culture supernatants were significantly lower in the subjects of rs5743867CC genotype than in CT and TT genotype subjects (*P *= 0.016 and *P *= 0.003 for TNF-α; *P *= 0.01 and *P *= 0.002 for IL-6, respectively).

**Conclusions:**

Our findings indicated that the variants in *TOLLIP *were significantly associated with sepsis susceptibility in the Chinese Han population.

## Introduction

Despite continuous progress in the development of antibiotics and other supportive care therapies, sepsis remains an unconquered challenge for clinicians and has an unacceptably high mortality rate of 30% to 50% for severe sepsis and septic shock [1,2]. The pathophysiology of sepsis involves highly complex interactions between invading microorganisms, the innate and adaptive immune systems of the host, and multiple downstream events leading to organ dysfunction [3]. Numerous studies have suggested that individuals vary in their responses to infection [4]. Currently, more and more evidence shows that common genetic variants of the innate and adaptive immune response pathway genes play an important role in determining the susceptibility to and outcome of sepsis [5-10].

Toll-like receptors (TLRs), a family of immune receptors, were recently reported to be involved in the recognition of pathogen-associated molecular patterns and the initiation of host immune responses [11]. In humans, more than 10 functional TLRs have been identified [12]. Among them, TLR2, TLR4, and TLR9 have been established to play a key role in the mediation of systemic responses to invading pathogens during sepsis [11,12]. After recognition of their respective ligands, TLRs induce inflammatory reactions by the activation of signaling pathways mediated by the adapter proteins myeloid differentiation factor 88 (MyD88) and Toll/interleukin-1 (IL-1)-receptor domain-containing adapter-inducing interferon [12]. The immune response initiated by TLRs is an important mechanism of defense against pathogenic microorganisms. However, prolonged and excessive activation of TLR signaling pathways contributes to the pathogenesis of sepsis and organ injury. TLR signaling and subsequent functions, therefore, must be under tight negative regulation to maintain immune response balance [13]. Recent studies have indicated that several negative regulators of TLR signaling pathways, including Toll-interacting protein (TOLLIP), inhibited TLR signaling pathway-mediated inflammatory responses and restored immune system balance. Inadequate production of these endogenous negative regulators may also contribute to the pathogenesis of sepsis [14].

Several single-nucleotide polymorphisms (SNPs) in the TLR signaling pathway genes have been reported to influence the production of inflammatory cytokines and be associated with susceptibility to inflammatory diseases [15]. In studies focusing on infection or sepsis, associations have been described for SNPs in the *TLR1 *(rs5743551), *TLR2 *(rs5743708), *TLR4 *(rs4986790 and rs4986791), *TLR9 *(rs5743836), *IRAK1 *(rs1059703), and *TIRAP *genes (rs8177374 and rs7932766) [7,16-21]. However, no studies have addressed the impact of genetic variants in TLR signaling pathways and negative regulators on sepsis susceptibility in the Chinese Han population.

Therefore, given the pivotal role of TLR signaling pathways and their negative regulators in the development of sepsis, we hypothesized that variants in genes encoding components of the TLR signaling pathways and their negative regulators might confer susceptibility to sepsis. To test this hypothesis, we conducted a case control study using a tag SNP approach to investigate the association of variants in *TLR2*, *TLR4*, *TLR9*, *MyD88*, and *TOLLIP *with susceptibility to sepsis in the Chinese Han population. In addition, we performed functional evaluation of the associated SNP.

## Materials and methods

### Study design and enrollment

The diagnosis of sepsis met the criteria recommended by the American College of Chest Physicians and the Society of Critical Care Medicine Consensus Conference [22]. Severe sepsis was defined as sepsis in combination with sepsis-induced acute organ dysfunction in at least one organ. Acute organ dysfunction was defined as Sequential Organ Failure Assessment (SOFA) scores of more than 2 for the organ in question. The SOFA score was calculated daily. Clinical and demographic data at baseline, including Acute Physiology and Chronic Health Evaluation (APACHE) II scores, previous health status, source of infection, microbiology, and intensive care unit mortality, were obtained after the patient met severe sepsis criteria. Exclusion criteria included age below 18 years, pregnancy, severe chronic respiratory disease, severe chronic liver disease (defined as a Child-Pugh score of greater than 10), malignancy, use of high-dose immunosuppressive therapy, and AIDS. Sex- and age-matched controls were selected from healthy blood donors. Healthy controls were defined as individuals without any recent acute illness, any chronic illness, or a history of sepsis. To reduce the potential confounding from ethnic backgrounds, only the Han Chinese population was enrolled in this study. The study was approved by the ethics committee of Zhongshan Hospital of Fudan University (Shanghai, China) (record number 2006-23). Informed consent was obtained from subjects or from their legal surrogates before enrollment.

### Single-nucleotide polymorphism selection and genotyping

A total of five candidate genes involved in TLR signaling pathways and their negative regulators were selected on the basis of known biological activity: *TLR2*, *TLR4*, *TLR9*, *MyD88*, and *TOLLIP*. Tag SNPs were selected on the basis of the data of the Chinese Han in Beijing (CHB) from the HapMap project phase II [23]. Tag SNPs for each of the genes were selected separately. In total, 12 tag SNPs in the five genes were selected by Tagger within Haploview using the following tagging criteria: pairwise tagging of the HapMap population with *r*^*2 *^of at least 0.8 and a minor allele frequency (MAF) of at least 5%. Location and characterization of all of the tested SNPs are listed in Table 1.

**Table 1:** Characteristics of the genotyped single-nucleotide polymorphisms in the genes of Toll-like receptor signaling pathways and negative regulators

Gene	Location	SNP	SNP type	Major/minor allele	MAF	HWE *P *value
*TLR2*	4q32	rs1898830	Tag SNP, intron	A/G	0.45	0.35
		rs3804099	Tag SNP, exon	T/C	0.32	0.64
*TLR4*	9q32-q33	rs2149356	Tag SNP, intron	G/T	0.39	0.76
		rs11536879	Tag SNP, intron	A/G	0.16	0.47
		rs1927907	Tag SNP, intron	C/T	0.24	1.00
*TLR9*	3p21.3	rs352140	Tag SNP, exon	G/A	0.38	1.00
*MyD88*	3p22	rs7744	Tag SNP, 3' UTR	A/G	0.38	0.41
		rs6853	Tag SNP, 3' UTR	A/G	0.01	1.00
*TOLLIP*	11p15.5	rs3750920	Tag SNP, exon	G/A	0.28	0.57
		rs5743867	Tag SNP, intron	T/C	0.35	0.61
		rs3793964	Tag SNP, intron	A/G	0.37	0.07
		rs3793963	Intron	G/A	0.25	0.30
		rs5744002	Intron	G/A	0.33	1.00
		rs5743942	Tag SNP, intron	T/C	0.12	1.00
		rs5743944	Intron	G/A	0.26	0.87
		rs5743947	Intron	G/A	0.31	0.53

HWE, Hardy-Weinberg equilibrium; MAF, minor allele frequency; MyD88, myeloid differentiation factor 88; SNP, single-nucleotide polymorphism; TLR, Toll-like receptor; TOLLIP, Toll-interacting protein; UTR, untranslated region.

Genomic DNA was extracted from whole blood with a FlexiGene DNA Kit (Qiagen, Hilden, Germany) in accordance with the protocol of the manufacturer. Genotyping was performed by direct sequencing. The sequencing reactions were performed with Applied Biosystems BigDye (version 3.1) chemistry (Applied Biosystems, Foster City, CA, USA), and the sequences were resolved with an ABI 3730 Genetic Analyzer. The primers and polymerase chain reaction (PCR) protocols used are shown in Table S1 in Additional file 1. Analyses of the sequence traces were performed with the Staden package and double-scored by a second operator.

### Isolation and stimulation of cells from healthy subjects

Peripheral blood mononuclear cells (PBMCs) were derived from healthy subjects by means of the Ficoll gradient density centrifugation method. Isolated PBMCs were plated at a density of 1 × 10^6 ^cells per milliliter in 24-well plates and cultured in RPMI 1640 medium with 10% fetal bovine saline at 37°C with 5% CO_2_. The cells were incubated for 6 hours in the presence or absence of 100 ng/mL *Escherichia coli *0111:B4 lipopolysaccharide (LPS) (Sigma-Aldrich, St. Louis, MO, USA). After incubation, supernatants and cell pellets were harvested and stored at -80°C until use.

### RNA purification and *TOLLIP *mRNA expression analysis

Total RNA was extracted with an RNeasy Mini kit (Qiagen). One hundred nanograms of RNA was used for cDNA synthesis with a High-Capacity cDNA Reverse Transcription Kit (Applied Biosystems) in accordance with the protocol of the manufacturer. The synthesized cDNA was used for real-time PCR performed by SYBR green-based assay on an ABI 7900HT system (Applied Biosystems). The primers for the *TOLLIP *gene were forward 5'-CGGTGTACATCGGTGAGC-3' and reverse 5'-CGTCTCGTACACCGCGTAG-3'. The primers for the endogenous control gene glyceraldehyde-3-phosphate dehydrogenase (*GAPDH*) were forward 5'-AAGGTCGGAGTCAACGGATT-3' and reverse 5'-CTCCTGGAAGATGGTGATGG-3'. We carried out initial denaturation at 95°C for 10 seconds followed by 40 cycles of PCR (95°C for 5 seconds, 57°C for 30 seconds, and 72°C for 30 seconds). *TOLLIP *mRNA expression levels were normalized to the levels of *GAPDH*. All experiments were run in triplicate. Independent cDNA synthesis was carried out twice.

### Measurement of tumor necrosis factor-alpha and interleukin-6 levels

Concentrations of tumor necrosis factor-alpha (TNF-α) and IL-6 in culture supernatants were measured with a human enzyme-linked immunosorbent assay (ELISA) kit (R&D Systems, Inc., Minneapolis, MN, USA) in accordance with the protocol of the manufacturer.

### Statistical analysis

The demographic variables between different groups were compared by chi-square test for categorical variables. The genotype data were analyzed for deviations from Hardy-Weinberg equilibrium by the Haploview version 4.1 software [24]. The differences of allele and genotype distributions between the sepsis and healthy control groups were compared with the chi-square test or Fisher's exact test when appropriate. *P *values for genotypic distributions were calculated with the global genotype test. Allele frequencies of cases and controls were used to calculate the odds ratio (OR) and the 95% confidence interval (CI). Multivariate logistic regression was used to adjust for potential confounding factors, including age and gender. Block was determined by Haploview version 4.1 with a linkage disequilibrium (LD)-based partitioning algorithm [25]. The data of the observed blocks were analyzed with the omnibus test and haplotype-specific association statistics (T test) as implemented in PLINK [26]. The case/control omnibus test was an H-1 degree of freedom test, in which H was the number of different haplotypes. The Bonferroni method was used to correct for multiple comparisons where applicable. A two-tailed *P *value of less than 0.05 was considered statistically significant, whereas a value of corrected *P *of less than (0.05 divided by the number of tests) was considered significant after Bonferroni correction. Differences in relative mRNA expression and TNF-α and IL-6 levels between genotypes were evaluated by one-way analysis of variance (ANOVA). When a significant difference was obtained in ANOVA, *post hoc *comparison with the least significant difference test was used to identify specific group differences. The software used for statistical calculations was the SPSS 15.0 (SPSS, Inc., Chicago, IL, USA) unless specified otherwise.

## Results

### Characteristics of the study population

From February 2006 to November 2009, 378 patients with severe sepsis were enrolled in this case control study. An additional population of 390 ethnicity-matched healthy volunteers was recruited for comparison. The baseline characteristics and clinical data of all subjects are shown in Table 2. The mean ages were 64.1 years for patients with severe sepsis and 65.8 years for healthy controls (*P *> 0.05). The proportions of males were 58.2% in patients with severe sepsis and 57.9% in healthy controls (*P *> 0.05). The primary sources of infection were the lungs (85.4%), followed by abdomen (6.1%), blood stream (3.2%), urinary tract (2.9%), and others (2.4%). The overall 30-day mortality rate of patients with severe sepsis was 32.3%.

**Table 2:** Demographic and clinical characteristics of the study subjects

	Healthy controls	Patients with sepsis
Number	390	378
Age, years	65.8 ± 12.2	64.1 ± 12.6
Males/Females	226/164	220/158
APACHE II score	NA	18.3 ± 4.3
Survival	NA	67.7%
Length of ICU stay, days	NA	18.6 ± 5.6
Diabetes	NA	39 (10.3%)
Chronic liver disease	NA	12 (3.2%)
Chronic renal failure	NA	16 (4.2%)
Congestive heart failure	NA	23 (6.1%)
Chronic pulmonary disease	NA	31 (8.2%)
Sepsis insult		
Lung	NA	323 (85.4%)
Abdomen	NA	23 (6.1%)
Bloodstream	NA	12 (3.2%)
Urinary tract infection	NA	11 (2.9%)
Others	NA	9 (2.4%)
Microbiology positive	NA	159 (42.1%)
Gram-positive	NA	61 (38.4%)
Gram-negative	NA	65 (40.9%)
Fungi	NA	15 (9.4%)
Mixed	NA	18 (11.3%)
Microbiology unknown	NA	219 (57.9%)

APACHE II, Acute Physiology and Chronic Health Evaluation II; ICU, intensive care unit; NA, not applicable.

### Association analyses of *TLR2*, *TLR4*, *TLR9*, *MyD88*, and *TOLLIP *polymorphisms with susceptibility to sepsis

All of the 12 tag SNPs were genotyped successfully by direct sequencing. Four other SNPs located in the intron region of *TOLLIP *(rs3793963, rs5744002, rs5743944, and rs5743947) were identified in the process of sequencing (Table 1). The genotyping success rates ranged from 97.5% to 99%, and all of the genotype distributions were consistent with Hardy-Weinberg equilibrium (*P *> 0.05) (Table 1). The allele and genotype distributions of these SNPs in healthy controls and patients with sepsis are listed in Table 3 and in Table S2 in Additional file 1. When patients with sepsis were compared with healthy controls, two tag SNPs in *TOLLIP *were observed in association with sepsis susceptibility. The minor allele C of rs5743867 in *TOLLIP *was associated with a decreased risk of sepsis (*P *= 0.00016, OR = 0.67, 95% CI 0.54 to 0.82), and the significance remained present after Bonferroni correction (*P *= 0.0026 corrected for 16 SNPs tested). Furthermore, in multivariate logistic analyses adjusting for age and gender, the rs574386 C allele was still significantly associated with protection from sepsis (*P*_adj _= 0.00062, OR_adj _= 0.71, 95% CI 0.59 to 0.86). The genotype distribution of rs5743867 was also significantly different between sepsis and control groups (*P *= 0.001), and the difference remained significant after adjustment for age and gender in multiple logistic regression analysis (*P*_adj _= 0.0018) and for multiple comparisons (*P *= 0.016 corrected for 16 SNPs tested). SNP rs5743942 of *TOLLIP *also showed an association with sepsis susceptibility. The C allele of rs5743942 was associated with increased risk of sepsis (*P*_adj _= 0.034, OR_adj _= 1.40, 95% CI 1.03 to 1.88). Also, the genotype distribution was significantly different between sepsis and control groups (*P*_adj _= 0.016). However, the difference was not significant after Bonferroni correction (*P *> 0.05 corrected for 16 SNPs tested). Both allele and genotype distributions of the other 14 SNPs in *TLR2*, *TLR4*, *TLR9*, *MyD88*, and *TOLLIP *did not vary significantly between sepsis patients and healthy controls (Table S2 in Additional file 1). Because TLRs detect specific microbial components, we performed the association analyses of *TLR2 *and *TLR9 *with Gram-positive sepsis patients and *TLR4 *and *TLR9 *with Gram-negative sepsis patients. However, no significant difference was found (Tables S3 and S4 in Additional file 1).

**Table 3:** Association analysis of the eight single-nucleotide polymorphisms in *TOLLIP *between sepsis patients and healthy control subjects

			Allelic comparison				Genotypic comparison	
SNP	Control	Sepsis	*P*	***P***_**adj**_	OR (95% CI)	**OR**_**adj **_**(95% CI)**	*P*	***P***_**adj**_
rs3750920								
AA	29 (7.5%)	26(7.0%)						
AG	159 (41.3%)	160 (43.1%)						
GG	197 (51.2%)	185 (49.9%)	0.867	0.972	1.02 (0.82-1.27)	1.01 (0.72-1.38)	0.867	0.911
rs5743867								
CC	64 (16.6%)	32 (8.6%)						
CT	176 (45.7%)	161 (43.3%)						
TT	145 (37.7%)	179 (48.1%)	0.00016	0.00062	0.67 (0.54-0.82)	0.71 (0.59-0.86)	0.001	0.0018
rs3793964								
GG	39 (10.2%)	41 (11.1%)						
AG	196 (51.0%)	210 (56.6%)						
AA	149 (38.8%)	120 (32.3%)	0.140	0.251	1.17 (0.95-1.44)	1.09 (0.82-1.39)	0.179	0.280
rs3793963								
AA	18 (4.7%)	22 (5.9%)						
AG	151 (39.4%)	145 (39.1%)						
GG	214 (55.9%)	204 (55.0%)	0.635	0.664	1.06 (0.84-1.37)	1.04 (0.81-1.29)	0.752	0.794
rs5744002								
AA	42 (10.9%)	35 (9.5%)						
AG	161 (41.9%)	175 (47.4%)						
GG	181 (47.2%)	159 (43.1%)	0.591	0.694	1.06 (0.86-1.32)	1.02 (0.84-1.26)	0.752	0.810
rs5743942								
CC	6 (1.6%)	4 (1.1%)						
CT	65 (16.8%)	95 (25.5%)						
TT	315 (81.6%)	274 (73.4%)	0.021	0.034	1.45 (1.06-1.98)	1.40 (1.03-1.88)	0.013	0.016
rs5743944								
AA	19 (4.9%)	32 (8.7%)						
AG	156 (40.5%)	131 (35.4%)						
GG	210 (54.6%)	207 (55.9%)	0.607	0.642	1.06 (0.84-1.34)	1.03 (0.81-1.30)	0.074	0.102
rs5743947								
AA	33 (8.6%)	34 (9.2%)						
AG	157 (41.0%)	177 (47.8%)						
GG	193 (50.4%)	159 (43.0%)	0.094	0.302	1.21 (0.97-1.50)	1.05 (0.85-1.31)	0.118	0.231

Data are presented as number (percentage) of subjects. *P *was determined using the chi-square test. *P *value adjusted for age and gender (*P*_adj_) came from multivariate logistic regression. A *P *value of less than 0.003 (0.05/16) was considered statistically significant after Bonferroni correction. Rs5743867 remained significant after Bonferroni correction. CI, confidence interval; OR, odds ratio; OR_adj_, odds ratio adjusted for age and gender; SNP, single-nucleotide polymorphism; TOLLIP, Toll-interacting protein.

### Association analyses of *TOLLIP*, *TLR2*, *TLR4*, *TLR9*, and *MyD88 *haplotypes with susceptibility to sepsis

**Figure 1:**
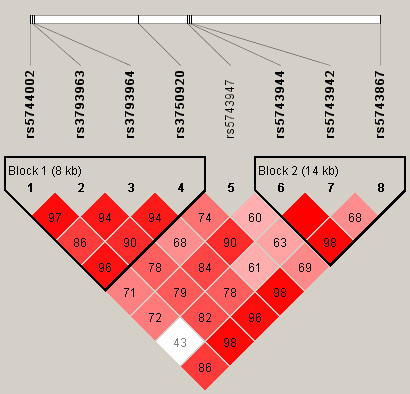
**Linkage disequilibrium (LD) plot of eight single-nucleotide polymorphisms in Toll-interacting protein (*TOLLIP*) genotyped in this study**. The plot was constructed with the Haploview program [24], and *r*^*2 *^(×100) values are depicted in the diamonds. Blocks were determined by Haploview with an LD-based partitioning algorithm [25]. The LD color scheme was stratified according to the logarithm of the odds (LOD) score and D': white, D' = 1 and LOD score = 2; pink or light red, D' = 1 and LOD score ≥2; and bright red, D' = 1 and LOD score ≥2.

We then performed haplotype analysis to investigate whether the haplotypes in the five genes were associated with sepsis risk. Two haplotype blocks in the *TOLLIP *region were determined by Haploview with an LD-based partitioning algorithm (Figure 1). Block 1 contained four SNPs (rs5744002, rs3793963, rs3793964, and rs3750920) spanning 8 kb on the upstream region of *TOLLIP*, which generated three common haplotypes with a frequency of greater than 5%: GGAG, AAGA, and GGGG. In the global test, haplotypes in this block were not significantly associated with sepsis risk (*P*_adj _= 0.244). The haplotype GGAG in this block was associated with decreased risk of sepsis with borderline significance (*P*_adj _= 0.041) (Table 4) but the association was not significant after correction for multiple testing. Block 2 harbored three SNPs (rs5743944, rs5743942 and rs5743867) spanning 14 kb on the downstream region of *TOLLIP*, which generated four haplotypes with a frequency of greater than 5%: GTC, GTT, ATT, and GCT. A global test showed a significant difference between sepsis and control groups, with a *P*_adj _value of 0.00049. Among these haplotypes, the haplotype GTC appeared protective and the frequency in the sepsis group was lower than in the healthy control group (*P*_adj _= 0.0012, OR_adj _= 0.73, 95% CI 0.62 to 0.89) (Table 4). Another haplotype, GCT, was significantly associated with increased risk of sepsis, and carriers of the GCT haplotype had a 1.62-fold increased risk for sepsis (*P*_adj _= 0.00092). No haplotypes in *TLR2*, *TRL4*, *TLR9*, and *MyD88 *were associated with sepsis risk in this study (data not shown).

**Table 4:** Associations between *TOLLIP *haplotypes and sepsis susceptibility

		Frequency					
LD block	**Haplotype**^**a**^	Healthy control	Sepsis	*P *value	***P***_**adj **_**value**	OR (95% CI)	**OR**_**adj **_**(95% CI)**
Block 1^b^	Global test			0.127	0.244		
	GGAG	0.615	0.558	0.027	0.041	0.79 (0.65-0.97)	0.86 (0.72-0.98)
	AAGA	0.225	0.215	0.636	0.768	0.94 (0.74-1.20)	0.98 (0.80-1.44)
	GGGG	0.060	0.083	0.088	0.177	1.41 (0.95-2.10)	1.03 (0.80-2.32)
Block 2^c^	Global test			0.00018	0.00049		
	GTC	0.380	0.299	0.00085	0.0012	0.69 (0.56-0.86)	0.73 (0.62-0.89)
	GTT	0.283	0.302	0.399	0.424	1.10 (0.88-1.38)	1.06 (0.80-1.34)
	ATT	0.248	0.261	0.590	0.778	1.07 (0.85-1.35)	1.02 (0.79-1.32)
	GCT	0.076	0.134	0.00028	0.00092	1.87 (1.33-2.63)	1.62 (1.27-2.86)

A *P *value of less than 0.013 (0.05/4) was considered statistically significant after Bonferroni correction. Haplotype GTC and GCT in block 2 remained significant after Bonferroni correction. ^a^Haplotype frequencies of less than 5% were not included in the analyses; ^b^the order of polymorphisms was rs5744002, rs3793963, rs3793964, and rs3750920; ^c^the order of polymorphisms was rs5743944, rs5743942, and rs5743867. CI, confidence interval; LD, linkage disequilibrium; OR, odds ratio; OR_adj_, odds ratio adjusted for age and gender; *P*_adj_, *P *value adjusted for age and gender; TOLLIP, Toll-interacting protein.

### Association analyses of *TOLLIP *mRNA expression level with rs5743867 genotypes

**Figure 2:**
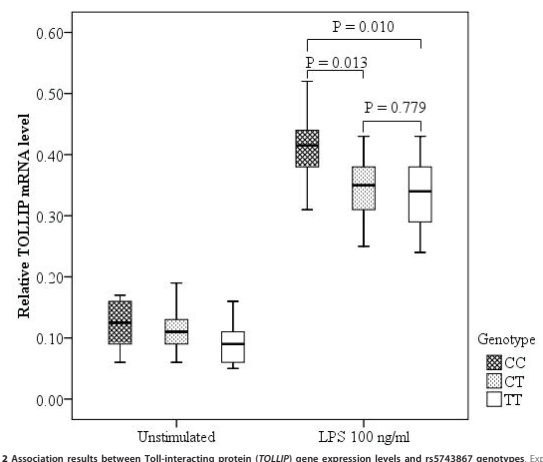
**Association results between Toll-interacting protein (*TOLLIP*) gene expression levels and rs5743867 genotypes**. Expression levels of *TOLLIP *in peripheral blood mononuclear cells were normalized with glyceraldehyde-3-phosphate dehydrogenase (*GAPDH*) expression and are presented as the median, interquartile range, and extremes. The mRNA expression levels of *TOLLIP *were significantly different among CC, CT, and TT genotypes under the lipopolysaccharide (LPS)-stimulated condition (*P *= 0.023, analysis of variance). No significant difference in *TOLLIP *mRNA expression levels was observed among CC, CT, and TT genotypes under the unstimulated condition (*P *= 0.156, analysis of variance).

We then evaluated the association between rs5743867 genotype and *TOLLIP *mRNA expression to determine whether the above SNP association reflected cis-acting regulatory effects on *TOLLIP*. A total of 38 healthy subjects were enrolled to determine the amount of *TOLLIP *mRNA expression level: 6 subjects with rs5743867CC genotype, 18 subjects with rs5743867CT genotype, and 14 subjects with rs5743867TT genotype. As shown in Figure 2, no significant difference in *TOLLIP *mRNA expression was observed among CC, CT, and TT genotypes in the unstimulated PBMCs (*P *> 0.05). After stimulation with LPS for 6 hours, the *TOLLIP *mRNA expression in PBMCs was significantly higher in CC homozygotes compared with both CT heterozygotes and TT homozygotes (*P *= 0.013 and *P *= 0.01, respectively), whereas the difference between the CT and TT groups was not statistically significant (*P *= 0.779).

### Association analyses of tumor necrosis factor-alpha and interleukin-6 levels with rs5743867 genotypes

**Figure 3:**
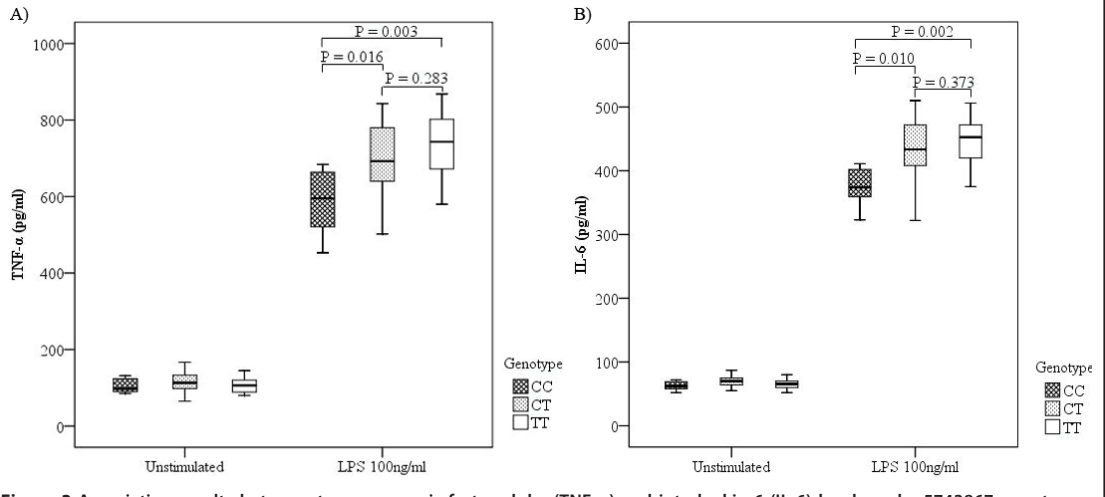
**Association results between tumor necrosis factor-alpha (TNF-α) and interleukin-6 (IL-6) levels and rs5743867 genotypes**. Concentrations of TNF-α and IL-6 in culture supernatants are presented as the median, interquartile range, and extremes. The TNF-α and IL-6 levels were significantly different among CC, CT, and TT genotypes under the lipopolysaccharide (LPS)-stimulated condition (*P *= 0.01, *P *= 0.012, analysis of variance). No significant difference in TNF-α and IL-6 levels was observed among CC, CT, and TT genotypes under the unstimulated condition (*P *= 0.528, *P *= 0.209, analysis of variance).

Because *TOLLIP *is involved in the cytokine processing, we also evaluated whether the variant influences TNF-α and IL-6 production (Figure 3). We observed a significant association between TNF-α and IL-6 levels and rs5743867 genotypes under the LPS-stimulated condition. Subjects with homozygotes for the rs5743867C allele were associated with lower levels of TNF-α and IL-6 compared with heterozygotes and homozygotes for the rs5743867T allele after LPS stimulation (*P *= 0.016 and *P *= 0.003 for TNF-α; *P *= 0.01 and *P *= 0.002 for IL-6, respectively). However, no significant association was observed between TNF-α and IL-6 levels and rs5743867 genotype under the unstimulated condition (*P *> 0.05).

## Discussion

This was the first report on genetic association analysis of TLR signaling pathways and their negative regulatory genes in Chinese Han patients with sepsis. Sixteen SNPs in five genes were successfully genotyped in this study. Our results showed that a tag SNP rs5743867 in *TOLLIP*, which influences the expression of *TOLLIP *mRNA and the production of TNF-α and IL-6, was significantly associated with susceptibility to sepsis. Consistent with the single SNP analyses, a three-SNP haplotype block harboring the associated SNP rs5743867 was also associated with the risk of sepsis.

The TLR signaling pathways and their negative regulators play a critical role in the pathogenesis of sepsis. Although several variants in the TLR signaling pathway genes have been implicated in susceptibility to sepsis and infectious diseases [7,16-20], the effect of variants in the negative regulatory genes of TLR signaling pathways on sepsis susceptibility has never been reported. We demonstrated here the first evidence for an association of sepsis susceptibility with variants in *TOLLIP*. TOLLIP, a negative regulator affecting cytoplasmic signal transduction, is widely expressed in a variety of human tissues. The inhibitory action of TOLLIP is mediated via suppression of autophosphorylation and kinase activity of IL-1 receptor-associated kinase 1, which is an important mediator in the TLR signal transduction [27]. Transfection of *TOLLIP *in intestinal epithelial cells resulted in decreased responsiveness to stimulation with LPS and lipoteichoic acid. Moreover, the production of inflammatory cytokines in *TOLLIP*-deficient mice, in comparison with that of wild-type mice, was significantly reduced [28].

The *in vitro *expression assays of mRNA and production of TNF-α and IL-6 in PBMCs under the LPS-stimulated condition clarified the functional relevance of SNP rs5743867 in *TOLLIP*. Subjects who were homozygotes with the C allele had higher mRNA expression of *TOLLIP *and lower levels of TNF-α and IL-6. These results indicated that SNP rs5743867 influenced the expression of *TOLLIP *and subsequently decreased the production of inflammatory cytokines. Rs5743867 is located in the intron region of *TOLLIP*. This is in accordance with the recent findings from genome-wide association studies that most of the associated variants of complex diseases are located outside the coding regions [29]. However, it is currently unclear how an intronic polymorphism can induce a phenotypic change. Rs5743867 may induce exon skipping, enhance the use of cryptic splice sites, or alter the ratio of alternatively spliced isoforms. Additionally, rs5743867 is more likely a marker in LD with a regulatory region polymorphism that controls expression levels of *TOLLIP *or a functional coding region SNP that influences the biological effect of *TOLLIP*. Exhaustive re-sequencing is needed to find or rule out the possibility of an as-yet-unidentified causal SNP in LD with rs5743867, and further functional evaluation of novel or associated SNPs is also needed.

To our knowledge, only two reports in the literature have described associations between *TOLLIP *variants and human diseases. Schimming and colleagues [30] demonstrated that the -526G/C (rs5743854) polymorphism in the promoter region of *TOLLIP *is significantly associated with the susceptibility of atopic dermatitis, which is a common inflammatory skin disorder. However, the mRNA expression of *TOLLIP *in lymphoid cells was not significantly different between the genotypes of rs5743854 [30]. Another study, conducted in 2008 by Wurfel and colleagues [7], screened SNPs in 43 TLR-related genes and identified one SNP (rs5743856) in *TOLLIP *affecting TLR-mediated inflammatory response. However, no study about the association between this functional polymorphism and sepsis susceptibility was reported. In our study, these two polymorphisms were not genotyped, because they were not included in the HapMap CHB data. Future study of *TOLLIP *should consider these functional variants.

Our results also indicated that tag SNPs of *TLR2*, *TLR4*, *TLR9*, and *MyD88 *did not represent major risk factors for sepsis development. Two nonsynonymous *TLR4 *SNPs (rs4986790 and rs4986791) have been shown to be associated with sepsis and infectious diseases in Caucasians and Africans. In another project (data not shown here), we observed that rs4986790 and rs4986791 are absent in Han Chinese populations, and this finding is in agreement with reports from other Asian populations [19,31,32]. Until now, no other SNPs or haplotypes of *TLR4 *were found to be associated with the susceptibility of sepsis or infectious diseases among Asian populations. It was reported that polymorphisms in *TLR2 *and *TLR9 *were associated with tuberculosis and other infectious diseases in previous studies; however, no association with sepsis susceptibility was found in our study [33,34]. One reason for these inconsistencies could be explained by the fact that the spectrum of infectious pathogens in our study was different from that of previous studies.

There were several limitations in our study. First, the association needs to be replicated in independent studies. Further replication studies in other populations are also expected. Second, we did not re-sequence the gene and instead used publicly available SNP databases. Thus, some variants could have been missed because of the incompleteness of these databases. Additionally, we did not evaluate whether the expression levels of *TOLLIP *are different between septic and non-septic patients.

## Conclusions

In our study, genetic and expression evidence indicated that a tag SNP in the intron region of *TOLLIP *was associated with sepsis susceptibility in the Chinese Han population by influencing the expression levels. These data supported the concept that genetic variation in the negative regulators of TLR signaling pathways plays an important role in the development of sepsis. Of note, whether the genetic variation is associated with sepsis susceptibility in other populations still needs to be explored.

## Key messages

• Individuals carrying the T allele of rs5743867 and haplotype GCT in Toll-interacting protein (*TOLLIP*) gene have a higher risk of developing sepsis in the Chinese Han population.

• Single-nucleotide polymorphism (SNP) rs5743867 influences the expression of *TOLLIP *mRNA and the production of tumor necrosis factor-alpha and interleukin-6.

• Tag SNPs of *TLR2*, *TLR4*, T*LR9*, and *MyD88 *are not associated with sepsis susceptibility in the Chinese Han population.

## Abbreviations

ANOVA: analysis of variance; CHB: Chinese Han in Beijing; CI: confidence interval; GAPDH: glyceraldehyde-3-phosphate dehydrogenase; IL: interleukin; LD: linkage disequilibrium; LPS: lipopolysaccharide; MyD88: myeloid differentiation factor 88; OR: odds ratio; OR_adj_: odds ratio adjusted for age and gender; *P*_adj_: *P *value adjusted for age and gender; PBMC: peripheral blood mononuclear cell; PCR: polymerase chain reaction; SNP: single-nucleotide polymorphism; SOFA: Sequential Organ Failure Assessment; TLR: Toll-like receptor; TNF-α: tumor necrosis factor-alpha; TOLLIP: Toll-interacting protein.

## Authors' contributions

CT headed the project and supervised and conducted the study. Z Song designed the study, carried out the statistical analysis, and drafted the manuscript. JY performed the data collection in the sepsis patient group and helped to conduct the experiments. CY, Z Sun, MS, YZ, and ZT were involved in the recruitment of the sepsis patients and healthy controls. PH participated in the study design and helped to draft the manuscript. All authors read and approved the final manuscript.

## Competing interests

The authors declare that they have no competing interests.

## Supplementary Material

Additional file 1 **Supplementary data**. A word document containing the following tables: Table S1: Primers and PCR protocols for SNPs genotyping; Table S2: Allele and genotype frequencies of *TLR2, TLR4, TLR9 *and *MyD88 *in the study subjects; Table S3: Allele and genotype frequencies of *TLR2 *and *TLR9 *in the gram-positive sepsis patients and healthy controls; Table S4: Allele and genotype frequencies of *TLR4 *and *TLR9 *in the gram-negative sepsis patients and healthy controls. Click here for file
