# Supplementary material for: Variants in the Toll-interacting protein gene are associated with susceptibility to sepsis in the Chinese Han population
Source: Crit Care. 2011 Jan 10;15(1):R12. doi: 10.1186/cc9413 (PMC3222042; doi:10.1186/cc9413)
Supplement: Additional file 1 — Supplementary data. A word document containing the following tables: Table S1: Primers and PCR protocols for SNPs genotyping; Table S2: Allele and genotype frequencies of TLR2, TLR4, TLR9 and MyD88 in the study subjects; Table S3: Allele and genotype frequencies of TLR2 and TLR9 in the gram-positive sepsis patients and healthy controls; Table S4: Allele and genotype frequencies of TLR4 and TLR9 in the gram-negative sepsis patients and healthy controls. [file cc9413-S1.DOC]

**Supplemental Table S1. Primers and PCR protocols for SNPs genotyping**

| Gene | Target Primer | Sequence (5'→3') | PCR protocol |
| --- | --- | --- | --- |
| TLR2 | rs1898830 |  | 95°C for 5 min; 35 cycles at 94°C for 30 s, 57°C for 40 s, |
|  |  |  | and 72°C for 45 s; followed by 72°C for 10 min |
|  | Forward | GCAGCTGAAATCACAGAGCA |  |
|  | Reverse | AGGATAATGGCCTCCTGCTT |  |
| TLR2 | rs3804099 |  | 95°C for 5 min; 35 cycles at 94°C for 30 s, 57°C for 40 s, |
|  |  |  | and 72°C for 45 s; followed by 72°C for 10 min |
|  | Forward | CAGAGGTGTGTGAACCTCCA |  |
|  | Reverse | ATATGCAGCCTCCGGATTGT |  |
| TLR4 | rs2149356 |  | 95°C for 5 min; 35 cycles at 94°C for 30 s, 57°C for 40 s, |
|  |  |  | and 72°C for 45 s; followed by 72°C for 10 min |
|  | Forward | TCCACAACGGTTCTCTGGAT |  |
|  | Reverse | AGCAAATAAGAAGCCCCACA |  |
| TLR4 | rs11536879 |  | 95°C for 5 min; 35 cycles at 94°C for 30 s, 57°C for 40 s, |
|  |  |  | and 72°C for 45 s; followed by 72°C for 10 min |
|  | Forward | CATGTTCTCTGCAATGGTTTG |  |
|  | Reverse | GCAGCAAGGGTTGAAAAACT |  |
| TLR4 | rs1927907 |  | 95°C for 5 min; 35 cycles at 94°C for 30 s, 57°C for 40 s, |
|  |  |  | and 72°C for 45 s; followed by 72°C for 10 min |
|  | Forward | TTCAACCCTTGCTGCTTTCT |  |
|  | Reverse | CGAAAAGGCAAAGGATGTCT |  |
| TLR9 | rs352140 |  | 95°C for 5 min; 35 cycles at 94°C for 30 s, 57°C for 40 s, |
|  |  |  | and 72°C for 45 s; followed by 72°C for 10 min |
|  | Forward | GGACACTCCCAGCTCTGAAG |  |
|  | Reverse | AAGGCCAGGTAATTGTCACG |  |
| MyD88 | rs7744 |  | 95°C for 5 min; 35 cycles at 94°C for 30 s, 57°C for 40 s, |
|  | rs6853 |  | and 72°C for 45 s; followed by 72°C for 10 min |
|  | Forward | GCACATGGGCACATACAGAC |  |
|  | Reverse | CCAGTGGACCAGCTTCTCTT |  |
| TOLLIP | rs3750920 |  | 95°C for 5 min; 35 cycles at 94°C for 30 s, 57°C for 40 s, |
|  |  |  | and 72°C for 45 s; followed by 72°C for 10 min |
|  | Forward | AGTAGAGAGCGCATGGGAAG |  |
|  | Reverse | GTGCTCCAACATACCCCAAT |  |
| TOLLIP | rs5743867 |  | 95°C for 5 min; 35 cycles at 94°C for 30 s, 57°C for 40 s, |
|  |  |  | and 72°C for 45 s; followed by 72°C for 10 min |
|  | Forward | CTGTTACACCCGCGATAGA |  |
|  | Reverse | CAACCCCTACTCCCATTCCT |  |
| TOLLIP | rs3793964 |  | 95°C for 5 min; 35 cycles at 94°C for 30 s, 57°C for 40 s, |
|  | rs3793963 |  | and 72°C for 45 s; followed by 72°C for 10 min |
|  | rs5744002 |  |  |
|  | Forward | CAGCCAACTGCTCAATTTCC |  |
|  | Reverse | CCTGCGTGTTTTGCTTTCAT |  |
| TOLLIP | rs5743942 |  | 95°C for 5 min; 35 cycles at 94°C for 30 s, 57°C for 40 s, |
|  | rs5743944 |  | and 72°C for 45 s; followed by 72°C for 10 min |
|  | rs5743947 |  |  |
|  | Forward | CTTTCTCGTGGGCACTGTC |  |
|  | Reverse | GTGCTGTCTCTGCCTAGTG |  |

**Supplemental Table S2.** Allele and genotype frequencies of *TLR2*, *TLR4*, *TLR9* and *MyD88* in the study subjects

|  |  |  | Allelic Comparison | | | | Genotypic Comparison | |
| --- | --- | --- | --- | --- | --- | --- | --- | --- |
| SNP | Control | Severe Sepsis | *P* | *P*_adj_ | OR (95% CI) | OR_adj_ (95% CI) | *P* | *P*_adj_ |
| TLR2 |  |  |  |  |  |  |  |  |
| rs1898830 |  |  |  |  |  |  |  |  |
| GG | 99 (25.7%) | 76 (20.5%) |  |  |  |  |  |  |
| GA | 160 (41.6%) | 175 (47.2%) |  |  |  |  |  |  |
| AA | 126 (32.7%) | 120 (32.3%) | 0.344 | 0.592 | 0.91 (0.74-1.11) | 0.98 (0.79-1.29) | 0.167 | 0.326 |
| rs3804099 |  |  |  |  |  |  |  |  |
| CC | 41 (10.7%) | 45 (12.1%) |  |  |  |  |  |  |
| CT | 157 (40.9%) | 152 (40.9%) |  |  |  |  |  |  |
| TT | 186 (48.4%) | 175 (47.0%) | 0.557 | 0.668 | 1.07 (0.86-1.33) | 1.03 (0.82-1.28) | 0.814 | 0.901 |
| TLR4 |  |  |  |  |  |  |  |  |
| rs2149356 |  |  |  |  |  |  |  |  |
| TT | 62 (16.1%) | 57 (15.4%) |  |  |  |  |  |  |
| TG | 164 (42.7%) | 188 (50.8%) |  |  |  |  |  |  |
| GG | 158 (41.2%) | 125 (33.8%) | 0.188 | 0.416 | 1.15 (0.93-1.41) | 1.02 (0.81-1.34) | 0.066 | 0.201 |
| rs11536879 | |  |  |  |  |  |  |  |
| GG | 14 (3.6%) | 16 (4.3%) |  |  |  |  |  |  |
| AG | 82 (21.4%) | 94 (25.4%) |  |  |  |  |  |  |
| AA | 288 (75.0%) | 260 (70.3%) | 0.149 | 0.321 | 1.23(0.93-1.62) | 1.16(0.81-1.56) | 0.346 | 0.452 |
| rs1927907 |  |  |  |  |  |  |  |  |
| TT | 16 (4.2%) | 26 (7.0%) |  |  |  |  |  |  |
| CT | 137 (35.8%) | 138 (37.2%) |  |  |  |  |  |  |
| CC | 230 (60.0%) | 207 (55.8%) | 0.106 | 0.326 | 1.22(0.96-1.54) | 1.15(0.81-1.52) | 0.182 | 0.324 |
| TLR9 |  |  |  |  |  |  |  |  |
| rs352140 |  |  |  |  |  |  |  |  |
| AA | 45 (11.7%) | 54 (14.6%) |  |  |  |  |  |  |
| AG | 192 (50.1%) | 176 (47.7%) |  |  |  |  |  |  |
| GG | 146 (38.2%) | 139 (37.7%) | 0.505 | 0.519 | 1.07(0.87-1.32) | 1.01(0.83-1.22) | 0.490 | 0.512 |
| MyD88 |  |  |  |  |  |  |  |  |
| rs7744 |  |  |  |  |  |  |  |  |
| GG | 48 (12.5%) | 44 (11.9%) |  |  |  |  |  |  |
| AG | 198 (51.6%) | 191 (51.6%) |  |  |  |  |  |  |
| AA | 138 (35.9%) | 135 (36.5%) | 0.817 | 0.842 | 0.98(0.79-1.20) | 1.02(0.89-1.16) | 0.964 | 0.981 |
| rs6853 |  |  |  |  |  |  |  |  |
| AG | 8 (2.1%) | 9 (2.4%) |  |  |  |  |  |  |
| AA | 377 (97.9%) | 361 (97.6%) | 0.744 | 0.843 | 1.17(0.45-3.06) | 1.09(0.62-3.12) | 0.743 | 0.854 |

Data were no. (%) of subjects. SNP, single nucleotide polymorphism; OR, odds ratio; CI, confidence interval; *P*_adj_ and OR_adj_, adjustment for age and gender

**Supplemental Table S3.** Allele and genotype frequencies of *TLR2* and *TLR9* in the gram-positive sepsis patients and healthy controls

|  |  | Gram-positive | Allelic Comparison | | | | Genotypic Comparison | |
| --- | --- | --- | --- | --- | --- | --- | --- | --- |
| SNP | Control | severe sepsis | *P* | *P*_adj_ | OR (95% CI) | OR_adj_ (95% CI) | *P* | *P*_adj_ |
| TLR2 |  |  |  |  |  |  |  |  |
| rs1898830 |  |  |  |  |  |  |  |  |
| GG | 99 (25.7%) | 10 (16.9%) |  |  |  |  |  |  |
| GA | 160 (41.6%) | 32 (54.2%) |  |  |  |  |  |  |
| AA | 126 (32.7%) | 17 (28.9%) | 0.623 | 0.562 | 0.91 (0.61-1.34) | 0.92 (0.71-1.24) | 0.154 | 0.146 |
| rs3804099 |  |  |  |  |  |  |  |  |
| CC | 41 (10.7%) | 6 (10.2%) |  |  |  |  |  |  |
| CT | 157 (40.9%) | 24 (40.7%) |  |  |  |  |  |  |
| TT | 186 (48.4%) | 29 (49.2%) | 0.894 | 0.782 | 0.97 (0.64-1.48) | 0.94 (0.77-1.74) | 0.991 | 0.901 |
| TLR9 |  |  |  |  |  |  |  |  |
| rs352140 |  |  |  |  |  |  |  |  |
| AA | 45 (11.7%) | 10 (16.9%) |  |  |  |  |  |  |
| AG | 192 (50.1%) | 26 (44.1%) |  |  |  |  |  |  |
| GG | 146 (38.2%) | 23 (39.0%) | 0.650 | 0.571 | 1.10 (0.74-1.63) | 1.14 (0.71-1.68) | 0.472 | 0.442 |

Data were no. (%) of subjects. SNP, single nucleotide polymorphism; OR, odds ratio; CI, confidence interval; *P*_adj_ and OR_adj_, adjustment for age and gender

**Supplemental Table S4.** Allele and genotype frequencies of TLR4 and TLR9 in the gram-negative sepsis patients and healthy controls

|  |  | Gram-negative | Allelic Comparison | | | | Genotypic Comparison | |
| --- | --- | --- | --- | --- | --- | --- | --- | --- |
| SNP | Control | severe sepsis | *P* | *P*_adj_ | OR (95% CI) | OR_adj_ (95% CI) | *P* | *P*_adj_ |
| TLR4 |  |  |  |  |  |  |  |  |
| rs2149356 |  |  |  |  |  |  |  |  |
| TT | 62 (16.1%) | 8 (12.7%) |  |  |  |  |  |  |
| TG | 164 (42.7%) | 30 (47.6%) |  |  |  |  |  |  |
| GG | 158 (41.2%) | 25 (39.7%) | 0.831 | 0.768 | 0.96 (0.65-1.42) | 0.92 (0.61-1.44) | 0.691 | 0.662 |
| rs11536879 | |  |  |  |  |  |  |  |
| GG | 14 (3.6%) | 4 (6.5%) |  |  |  |  |  |  |
| AG | 82 (21.4%) | 16 (25.8%) |  |  |  |  |  |  |
| AA | 288 (75.0%) | 42 (67.8%) | 0.146 | 0.152 | 1.44 (0.88-2.34) | 1.39 (0.86-2.44) | 0.386 | 0.417 |
| rs1927907 |  |  |  |  |  |  |  |  |
| TT | 16 (4.2%) | 3 (4.8%) |  |  |  |  |  |  |
| CT | 137 (35.8%) | 26 (41.9%) |  |  |  |  |  |  |
| CC | 230 (60.0%) | 33 (53.2%) | 0.355 | 0.366 | 1.23 (0.79-1.90) | 1.31 (0.82-1.91) | 0.598 | 0.545 |
| TLR9 |  |  |  |  |  |  |  |  |
| rs352140 |  |  |  |  |  |  |  |  |
| AA | 45 (11.7%) | 11 (17.2%) |  |  |  |  |  |  |
| AG | 192 (50.1%) | 28 (43.8%) |  |  |  |  |  |  |
| GG | 146 (38.2%) | 25 (39.1%) | 0.626 | 0.588 | 1.10 (0.75-1.62) | 1.08 (0.81-1.61) | 0.415 | 0.410 |

Data were no. (%) of subjects. SNP, single nucleotide polymorphism; OR, odds ratio; CI, confidence interval; *P*_adj_ and OR_adj_, adjustment for age and gender
